# Outdoor Rearing and Behavioural Patterns in Diverse Rabbit Breeds: An Exploratory Study

**DOI:** 10.3390/ani15243562

**Published:** 2025-12-11

**Authors:** Luigia Bosa, Gloria Bernabucci, Francesca Di Federico, Lorenzo Nompleggio, Marta Vispi, Laura Menchetti, Alessandro Dal Bosco, Simona Mattioli, Riccardo Primi, Pedro Girotti, Cesare Castellini

**Affiliations:** 1 Department of Agricultural, Food and Environmental Sciences, University of Perugia, 06121 Perugia, Italy; francesca.difederico@unipg.it (F.D.F.); lorenzo.nompleggio@studenti.unipg.it (L.N.);; 2 Department of Agriculture and Forest Sciences, University of Tuscia, 01100 Viterbo, Italy; gloria.bernabucci@unitus.it (G.B.); primi@unitus.it (R.P.);; 3 School of Biosciences and Veterinary Medicine, University of Camerino, 62032 Camerino, Italy; laura.menchetti@unicam.it

**Keywords:** animal behaviour, free-range rabbit farming, breed differences, grazing behavior, organic rabbit production

## Abstract

EU regulations on organic rabbit farming are relatively recent, and scientific evidence on key technical aspects remains limited. Outdoor systems may improve health and welfare by allowing natural behaviours, but their effectiveness depends on management practices, environmental conditions, and breed. The objective of this study was to explore breed-related differences in rabbit behaviour under outdoor rearing conditions. A total of 15 Leprino di Viterbo (LV) and 15 New Zealand White (NZW) rabbits were weaned at 29 days of age and reared under outdoor conditions until 84 days of age, during October and November. All animals had ad libitum access to a commercial pelleted diet and pasture, and the intake of both was measured weekly. Animal behaviour was monitored by video recording, and eight one-minute sample intervals per day were analysed using focal sampling and continuous recording methods. Grass intake and estimated digestible energy (DE) were assessed on a weekly basis. Data were analysed using Generalized Estimating Equations to evaluate the effects of time, time of day, and breed. Behavioural patterns varied depending on genetic strain and time of day. Notably, LV rabbits exhibited a higher frequency of grazing and active behaviours compared to NZW rabbits, whereas NZW rabbits showed a higher frequency of resting behaviours and social contact. Breed-related differences in other behaviors and in grass intake patterns were not statistically robust. In conclusion, LV rabbits appear to be better adapted to outdoor conditions, exhibiting a greater pasture utilization, but further studies are recommended to confirm these findings and to evaluate their robustness across different seasonal and environmental conditions.

## Simple Summary

Outdoor rearing systems can improve rabbit welfare by allowing animals to move freely and express their natural behaviours. However, the response to these conditions may differ among breeds. This exploratory study provides a first comparison of the behaviour of a local Italian breed (Leprino di Viterbo) and the commercial New Zealand White, both reared outdoors from weaning to slaughter age. Their behaviour was recorded using video cameras and analysed to identify differences in activity and feeding patterns. Leprino rabbits showed higher levels of movement and grazing activity, while New Zealand White rabbits spent more time resting and in social contact. Both groups benefited from the outdoor environment, which allowed a wide expression of natural behaviours. Overall, the results suggest that the Leprino di Viterbo may be better suited to outdoor rearing, although further studies are needed to confirm this under different seasonal and management conditions.

## 1. Introduction

In Europe, the rabbit farming sector has long been in crisis due to declining rabbit meat consumption and growing societal concerns over conventional farming methods, particularly the use of cages. In fact, the current rearing system of rabbits in EU is mainly performed in cages, which does not permit the execution of the entire behaviour repertoire. Ethical considerations also play a role, as rabbits are often perceived as companion animals. On the other hand, rabbits exhibit a broad behavioural repertoire. They are social animals that require group living, opportunities to explore, run, jump, and, as prey animals, access to shelter and refuge [1,2,3]. Therefore, farmed rabbits should be allowed to express their natural behaviours, with sufficient space that mimics their wild counterparts, encouraging the adoption of less intensive farming practices [4].

Unlike hens, pigs, and calves, which are protected by specific European regulations, rabbits lack dedicated legislation governing their farming systems. Rabbit welfare is currently regulated only by general provisions, according to Council Directive 98/58/EC [5] of 20 July 1998 on the protection of animals kept for farming purposes (OJ L 221, 8 August 1998, pp. 23–27)”. Scientific opinions from bodies like the European Food Safety Authority (EFSA) offer guidance, but these are not legally binding. In recent years, the European citizens’ initiative End the Cage Age has garnered more than 1.4 million signatures calling for a ban on cage systems for laying hens, rabbits and other farm-animal species. Thus, the present study on outdoor rearing of rabbits is timely and aligns with broader policy and welfare debates. Only a few Member States have implemented specific measures: Austria has banned conventional cages; Belgium has transitioned entirely to “park” systems; Germany has issued non-binding national guidelines; and Italy’s Ministry of Health published voluntary guidelines in 2020. As a result, significant legislative gaps remain in the sector [6].

Despite these challenges, the rabbit sector warrants greater attention. Rabbits are highly efficient and sustainable source of protein, nutritionally valuable, and associated with a low environmental footprint [7]. As herbivores, rabbits do not compete directly with human food sources [8,9], unlike poultry, and they can convert grass into meat rich in long-chain (LC > C20) polyunsaturated fatty acids, which are beneficial to human health [10,11,12] Given these characteristics, the sector should be protected and revitalized through an integrated approach grounded in the principles of “One Welfare”. Accordingly, free-range and organic rabbit farming systems offer promising alternatives. Although organic rabbit farming is regulated within the EU, there remains a lack of robust scientific data and clear technical standards to support its practical implementation.

The transition towards outdoor and floor-based rabbit farming systems requires advances in husbandry practices, including the adoption of suitable breeds and the development of dedicated genetic improvement lines. Local breeds, typically more rustic and better adapted to “extensive” conditions than specialized meat lines such as the New Zealand White (NZW), may represent a promising option to enhance animal welfare and system sustainability. In this context, the Leprino di Viterbo (LV), a local Italian breed, was evaluated in comparison with the NZW with the aim of exploring potential breed-related differences in behavioural patterns and feeding strategies when reared in outdoor, ground-based paddocks. LV is a native rabbit breed from Central Italy, traditionally associated with small-scale and low-input farming systems. It is considered a rustic breed, well adapted to local environmental conditions and characterized by a moderate growth rate and a good ability to utilize natural vegetation. The New Zealand White, on the other hand, is a highly selected commercial breed that is widely used in intensive meat production due to its high productive performance. These differences in origin and selection history could influence how the two breeds respond to natural environmental stimuli, particularly in terms of physical activity, feeding behaviour and use of pasture.

To our knowledge, this study is the first exploratory assessment of post-weaning behaviour in these two rabbit breeds under outdoor conditions.

## 2. Materials and Methods

All methods were reported in accordance with the ARRIVE guidelines [13] for the reporting of animal experiments. The experimental design was developed in compliance with Italian directives on animal welfare for experimental and scientific purposes (Gazzetta Ufficiale, 26/2014 [14]) and was approved by the Ethics Committee of the University of Perugia (Protocol No. 349569, dated 17 October 2024).

At the end of the experimental period, after slaughtering the animals were examined to assess their general health condition. At the end of the experimental period, rabbits were transported to an authorised slaughter in accordance with Council Regulation (EC) No. 1099/2009 [15] on the protection of animals at the time of killing and the relevant national legislation. Animals were stunned prior to slaughter, following standard commercial procedures. Particular attention was given to organs commonly affected by *Eimeria* spp., including the liver, small intestine and cecum, which were inspected macroscopically to detect possible lesions related to coccidial infection.

### 2.1. Animal and Housing Conditions

**Figure 1:**
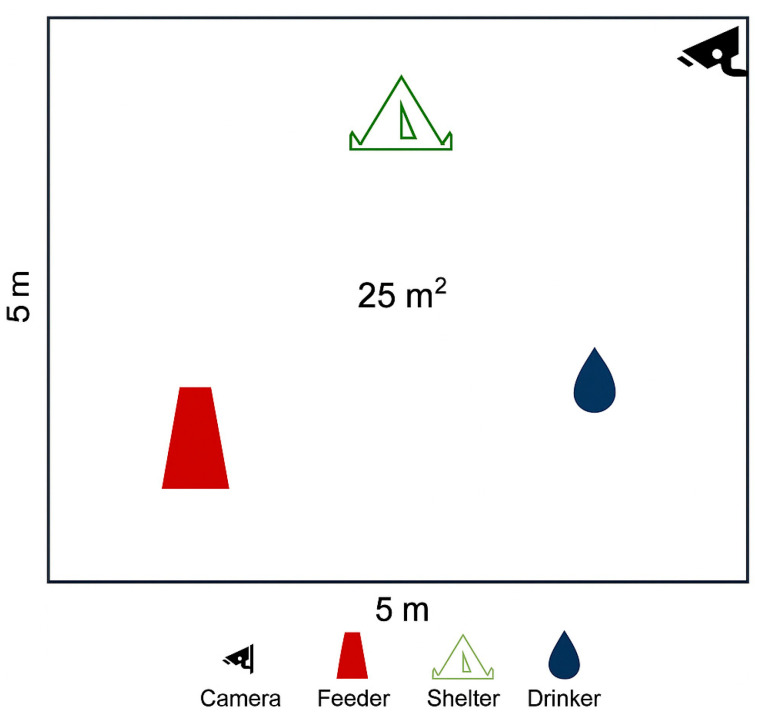
Diagram of the single experimental pen.

The trial was conducted between October and November 2024 at the ‘Nello Lupori’ experimental farm of the University of Tuscia (Viterbo, Italy; 42°25′36.1″ N, 12°4′49.3″ E). A total of 30 rabbits were used, comprising two genetic types: Leprino di Viterbo (LV; *n* = 15) and New Zealand White (NZW; *n* = 15), A total of 30 rabbits were used, comprising 15 Leprino di Viterbo and 15 New Zealand White, with a balanced sex ratio (50:50) in both breeds. All animals were weaned at 29 days of age (mean initial body weight of 435 + 30 and 500 + 38 g in LV and NZW, respectively) and subsequently randomly allocated to six outdoor paddocks (three per breed five rabbits per paddock). Each paddock measured 25 m^2^ (5 m^2^ per rabbit) and was equipped with a shelter, a feed trough, a drinker, and a Camera equipped with Passive InfraRed (PIR) sensors and day/night vision for behavioural monitoring. A schematic layout of the experimental paddock is shown in Figure 1.

The experimental design was based on the Italian transitional rules provided by Article 42 of European Regulation 834/2007 [16], which set 5 m^2^ as the minimum outdoor area per growing rabbit. Later on, EU (Commission Implementing Regulation (EU) 2020/464 [17]; Regulation 2018/848 [18]) and National rules (Ministerial Decree of 20 May 2022, No. 229771 [19]) established a minimum outdoor requirement of 0.5 m^2^ per rabbit.

The paddocks consisted of permanent mixed grassland, to which rabbits had ad libitum access, along with tap water and a commercial pelleted diet. The daily solid feed intake (g/d) was calculated by providing a known amount of feed in the trough and weighing the leftovers each day. Feed and grass samples were collected every six days for proximate analysis.

Grass intake for each paddock was estimated every six days using a modified version of the method described by [20] and the amount was expressed per day. This involved measuring the grass biomass at the beginning of the period, the growth in exclusion pens (0.50 × 0.50 m), and the residual grass at the end of the sub-period. The following equation was applied:

$$I=\left(GMs-GMe\right)+\frac{1-\left(GMe/GMs\right)}{-ln\left(GMe/GMs\right)}\left(GMu-GMs\right)$$

where:

- *GMs* = grass mass present before animals entered each paddock
- *GMe* = grass mass remaining at the end of the sub-period
- *GMu* = undisturbed forage mass from the exclusion pens

Grass in the pens and in the exclusion, pens was measured using a sward stick weekly (EC10 Electronic Plate Meter (Jenquip, Feilding, New Zealand)).

### 2.2. Environmental Conditions

**Figure 2:**
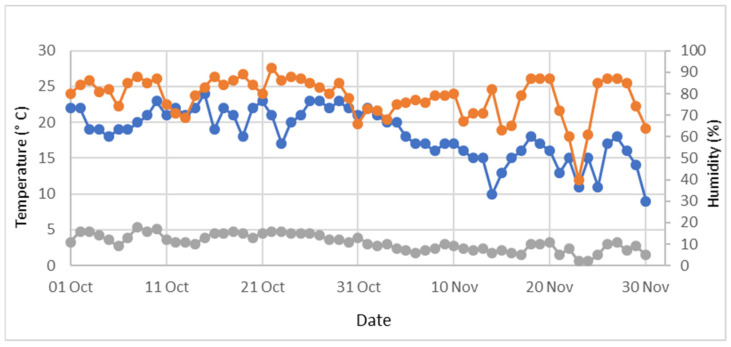
Daily minimum (blue line) and maximum (orange line) temperature (°C) and relative humidity (%; grey line) recorded during the trial period (1 October–30 November 2024).

The study area is characterised by a Mediterranean climate, with hot, dry summers and cool, rainy winters. The mean annual air temperature is approximately 14.5 °C, and the mean annual precipitation is 755 mm [21]. Environmental parameters, including daily minimum and maximum temperature and relative humidity, were recorded daily (Figure 2), along with wind intensity and rainfall, obtained from the meteorological service of Viterbo Airport station (Viterbo, Italy). During the trial, rainfall occurred on five days, and wind speeds exceeded 20 km/h on two days.

#### 2.2.1. Chemical Analysis of Feed and Grass

The dry matter (DM) content of feed and grass samples was determined by oven-drying at 65 °C until a constant weight was reached. Dried samples were then ground to pass through a 1 mm screen and analysed for crude protein [22], crude fibre [23], ether extract [24], and ash [22]. In addition, neutral detergent fibre (NDF), acid detergent fibre (ADF), acid detergent lignin (ADL) were determined using the filter bag system (Ankom Technology Corp., Fairport, NY, USA; [25]; hemicellulose and cellulose were also calculated. The chemical composition of the feed and grass used in the study is reported in Table 1.

Finally, digestible energy (DE) was estimated as described by [26] DE(MJ/kg DM) = 14.2 − 0.205 × ADF + 0.218 × EE + 0.057 × CP.

#### 2.2.2. Behavioural Data

Behavioural data were obtained from video recordings and analysed using the focal animal sampling method. Behavioural assessments were based on eight one-minute video recordings per day (four during the light phase and four during the dark phase). Each sampling interval was analysed five times to ensure the systematic observation of each individual rabbit. Observations were conducted every six days, beginning at 30 days of age (one day after weaning) and continuing until 84 days of age, resulting in ten observation periods. During each sampling interval, five rabbits were monitored, and the precise duration of each predefined behavioural category was recorded (Table 2). In total, the study generated 400 min of video recordings across the experimental period, which resulted in 2000 min of behavioural observations. The duration of each behaviour included in the ethogram (Table 2) was recorded and expressed as a percentage of the total time the animal was visible.

### 2.3. Statistical Analysis

Statistical analyses were performed on individual behaviours and on each behavioural category. Descriptive statistics were used to report the frequency of each observed behaviour. For inferential analysis, Generalized Estimating Equations (GEE) were used, applying a Tweedie distribution with a log link function to accommodate the nature of the behavioural data. Within-subject factors included age (i.e., Time; 10 levels), time of day (i.e., day/night; 2 levels), and scan within time of day (4 levels for daytime and 4 for night-time observations), modelled with an exchangeable correlation matrix to account for repeated measures. The models evaluated the main effects of Time, time of day, breed (2 levels: LV and NZW), and the interaction between Time and breed. To ensure robustness and relevance, only behaviours with a mean frequency of at least 1.0% in both breeds were included in the inferential analysis.

A non-parametric approach was employed to analyze grass intake, applying the Mann–Whitney test at each time point to compare the two breeds, and the Friedman test to evaluate within-breed changes over time.

Statistical analyses were performed with SPSS version 27 (IBM, SPSS Inc., Chicago, IL, USA). The level of statistical significance was set at *p* < 0.05.

## 3. Results

### 3.1. Effects of Circadian Timing, Age, and Breed on Individual Behaviours

**Figure 3:**
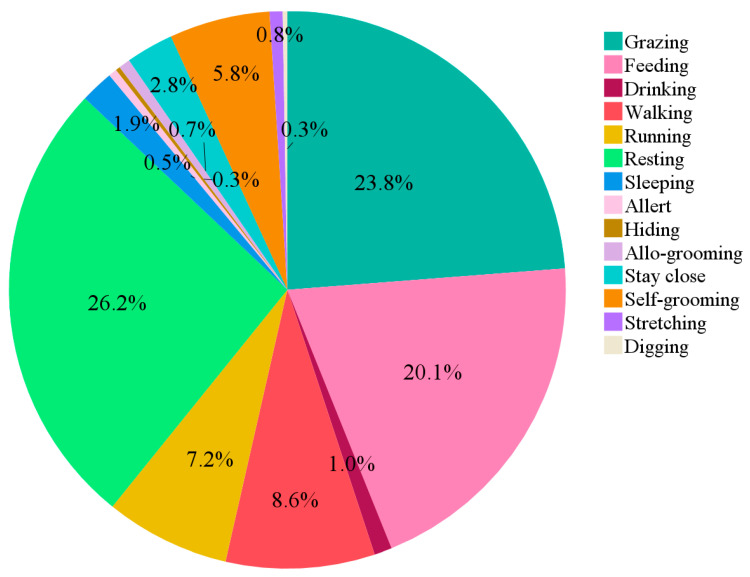
Pie chart showing of rabbit time budget regardless of breed, age, or time of day. Behaviours are expressed as a percentage of the total time the animal was visible.

The time budget of rabbits, regardless of breed, age, or time of day, is shown in Figure 3. Overall, the most frequently observed behaviours were resting, grazing, and feeding, followed by walking, running, self-grooming, staying close, sleeping, and drinking. The other behaviours were recorded with total frequencies below 1%.

Figure 3 presents the results of the inferential statistics, namely the marginal means for day and breed, the corresponding standard errors, and the outcomes of multiple comparisons (exact *p*-values are reported in Table S1).

#### 3.1.1. Ingestive Behaviours

**Figure 4:**
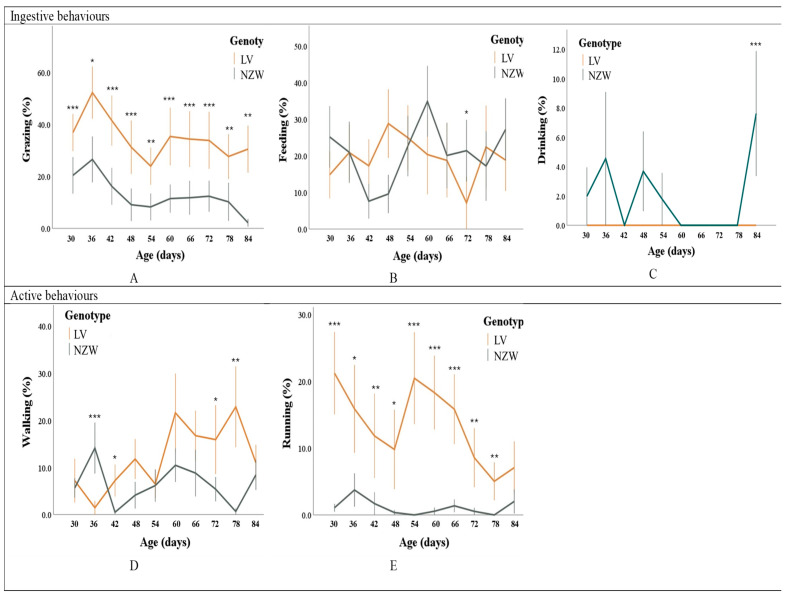

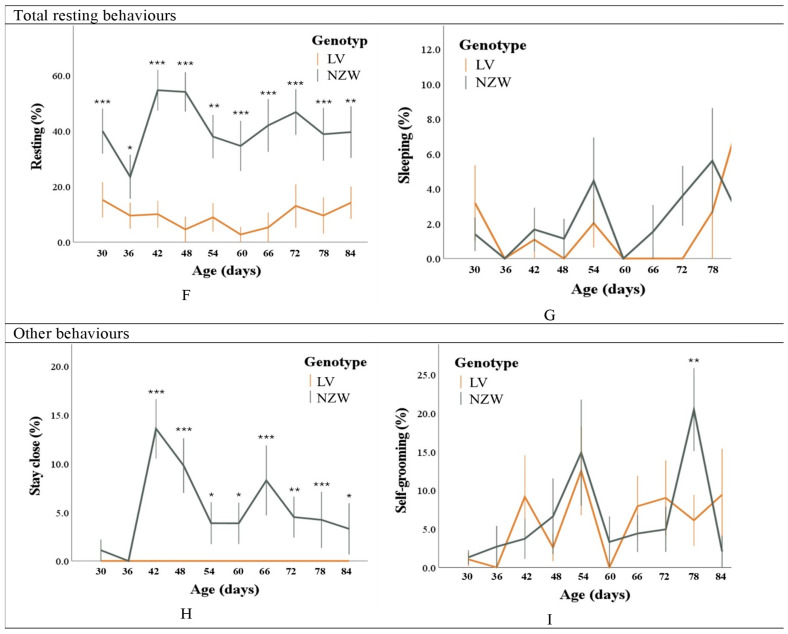
Mean frequencies per minute (and standard error) of the behaviours recorded in the two breeds from day 30 to day 84 of age. Behaviours are expressed as a percentage of the total time the animal was visible (**A**–**I**). Error bars that with asterisks are different at (*) *p* < 0.05; (**) *p* < 0.01; (***) *p* < 0.001, respectively. LV = Leprino di Viterbo, NZW = New Zealand White.

Grazing was significantly influenced by breed (*p* < 0.01), with LV rabbits exhibiting more than twice the frequency observed in NZW rabbits. Grazing behaviour also varied significantly over time (*p* < 0.001), showing a general downward trend in both breeds. However, the pattern differed between them (Time × Breed interaction: *p* < 0.001), as LV rabbits consistently maintained higher frequencies, falling below 20% (Figure 4A). Although no significant circadian rhythm was detected, descriptive data suggested a slight tendency for NZW rabbits to graze more during night-time compared to LV.

Feeding behaviour was significantly affected by age and by the interaction between age and breed (*p* < 0.001). New Zealand White rabbits exhibited a progressive reduction in feeding frequency during the first three weeks after weaning, followed by a peak on day 65. In contrast, LV rabbits displayed a more stable feeding pattern over the same period, with a marked decline on day 72, when their feeding frequency dropped below that of the NZW group (Figure 4B).

Drinking behavior was recorded only sporadically, which caused issues in applying the statistical model. Among the factors considered, it was influenced solely by the day (*p* < 0.001), with no differences between breeds (Figure 4C).

#### 3.1.2. Active Behaviours

Walking behaviour exhibited significantly higher marginal means in the LV breed (*p* < 0.001; Table S1), and distinct time-related patterns between breeds (*p* < 0.001; Figure 4E). Multiple comparisons revealed higher walking frequencies in the NZW breed on day 36, LV rabbits always surpassed them on subsequent days (*p* < 0.05; Figure 4D).

Running was significantly influenced by breed, age, and their interaction (*p* < 0.001), but not by time of day. LV rabbits showed marginal means for running frequency that were over ten times higher than those observed in NZW rabbits. LV rabbits consistently showed values above 5% across all observation days, while NZW rabbits never exceeded 4%, and the behaviour was entirely absent on several days (*p* < 0.05; Figure 4E).

#### 3.1.3. Total Resting Behaviours

Breed had a strong effect on resting behaviour (*p* < 0.001), with NZW rabbits resting approximately four times more frequently than LV ones. NZW rabbits consistently spent a greater proportion of time resting across all observation days, often exceeding 40%, whereas LV consistently showed lower frequencies, remaining below 20% and displaying no resting behaviour on certain days (Figure 4F).

Sleeping behaviour was influenced by age and its interaction with breed (*p* < 0.05). However, multiple comparisons did not reveal any significant differences between breeds on any specific observation day. This behaviour was completely absent on some days (Figure 4G).

#### 3.1.4. Other Behaviours

Among the other behavioural variables, only stay close and self-grooming reached frequencies as high as 1%. Inferential statistics were therefore applied only to these two behaviours, whereas for the remaining ones, only descriptive statistics were used (Table S1).

Due to the low frequency of occurrence, the model for ‘stay close’ did not estimate some effects. However, this behaviour was more expressed in NZW rabbits (*p* < 0.001) and during the day compared to the night (*p* < 0.05). Multiple comparisons further indicated higher frequencies in the NZW breed on most observation days (Figure 4H).

Self-grooming was affected by age and its interaction with breed (*p* < 0.001). Both breeds spent considerable time grooming on day 54 and very little on day 60. A significant difference between breeds was observed only on day 78, with NZW rabbits showing higher grooming frequencies than Leprino (*p* < 0.05; Figure 4I).

It is noteworthy that no aggressive behaviours were observed throughout the duration of the trial.

It should be underlined that no macroscopic alterations consistent with coccidiosis were observed in the liver, small intestine or caecum at post-mortem examination.

#### 3.1.5. Behavioural Traits Distinguishing the Two Breeds

**Figure 5:**
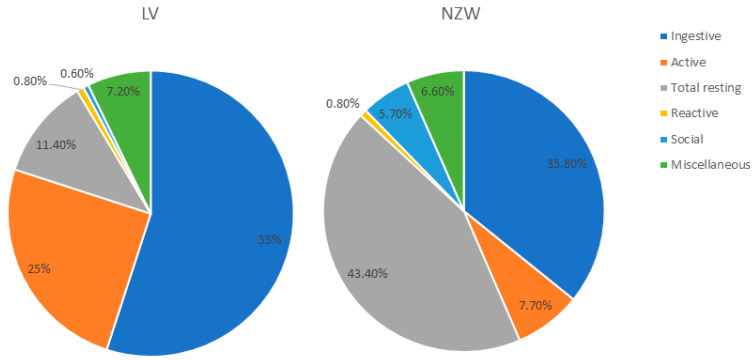
Relative frequencies (percentages per minute) of the behavioural categories recorded in the two breeds during the observational period (30–84 days of age). Behaviours are expressed as a percentage of the total time the animal was visible. Leprino= Leprino di Viterbo, NZW= New Zealand White.

Figure 5 presents the proportion of time spent in each behavioural category by rabbits of the two breeds throughout the observational period (30–84 days of age). In LV rabbits, ingestive and active behaviours predominated, collectively accounting 80% of total observed behaviours. These categories were significantly more frequent in LV rabbits compared to NZW (*p* < 0.001).

In contrast, resting behaviours were approximately four times more prevalent in NZW rabbits than in LV (*p* < 0.001), indicating a more sedentary behavioural profile.

Social behaviours also varied markedly between breeds, comprising roughly 6% of total behaviour in NZW rabbits but remaining below 1% in LV rabbits (*p* < 0.001).

No significant breed-related differences were observed for reactive behaviours (e.g., alertness and hiding), nor for maintenance and displacement behaviours.

### 3.2. DE Intake and (%) DE from Grass

**Figure 6:**
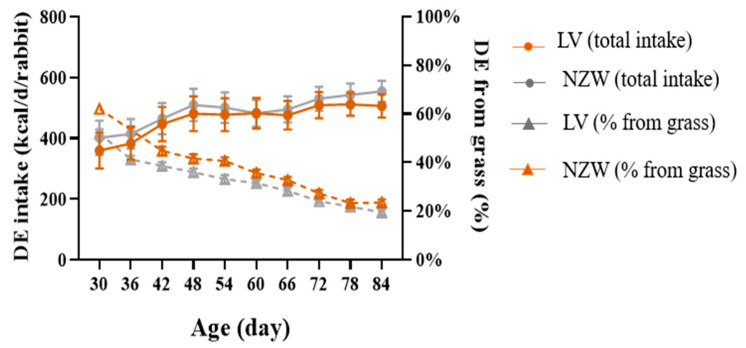
DE intake (left *y* axis) and % of DE obtained from grass (right *y* axis) in the two genetic lines (95% upper and lower limits). The values are means and standard errors of the three pens. LV = Leprino di Viterbo, NZW = New Zealand White.

Figure 6 shows the intake of DE and the percentage of DE derived from grass in the two breeds during the trial. Inferential analysis did not reveal any differences (*p* > 0.05) between breeds at any time point. Over time, total DE exhibited a progressive increase (*p* < 0.001), while the proportion of DE derived from grass showed a gradual decline in both breeds (*p* < 0.001).

## 4. Discussion

This study, although exploratory, suggests that rabbit breed can play a significant role in shaping behavioural patterns and adaptability in outdoor farming systems. Our findings indicate that although both breeds exhibited a broad behavioural repertoire, LV rabbits tended to display a more active and exploratory behavioural profile, characterized by higher frequencies of grazing, walking, and running. These traits may reflect a closer alignment of LV with species-specific ethology and could be associated with favourable welfare-related outcomes under free-range management. In contrast, NZW rabbits tended to exhibit more sedentary behaviour and a greater tendency to seek social contact, suggesting a different adaptive strategy that may be linked to production-oriented traits rather than environmental engagement. Rabbits kept in conventional cages usually have very limited space and few stimuli, and this inevitably affects their behaviour. In these systems, rabbits tend to spend most of their time resting, with reduced opportunities for movement and exploration. Indeed, the lack of environmental complexity and the absence of natural substrates, such as soil and vegetation, strongly limit the expression of natural behaviours.

On the contrary, outdoor systems provide a more stimulating environment and allow rabbits to move more freely and interact with a more complex space. This difference between housing systems helps to explain the greater expression of grazing and locomotor behaviours observed in the present study.

Despite these differences, both breeds benefited from the outdoor system, which facilitated the expression of species-specific behaviours. The observed behavioural diversity underscores the importance of tailoring management practices to the needs of different breeds, particularly within organic and free-range contexts [27]. Moreover, the observed decline in grazing activity over time underscores the importance of providing sufficient pasture area and implementing dynamic pasture management strategies, such as paddock rotation, to ensure consistent forage availability contemporary supporting the expression of natural behaviours.

Scientific evidence comparing different rabbit breeds under outdoor conditions remains very limited. This scarcity may be partly explained by the fact that the development of experimental protocols under free-ranging conditions involves substantial logistical, spatial, and economic challenges [28,29], which in our study resulted in the use of a limited number of replicates. For this reason, these results should be interpreted with caution. Accordingly, we deliberately chose to discuss only those findings supported by the strongest statistical evidence (i.e., ingestive and locomotor behaviours). In general, however, irrespective of the production system, the scientific literature still contains a limited number of studies comparing the behaviour of different rabbits. Ozella et al. [30] reported that selected breeds, such as NZW, reared in single cages or in groups, tend to exhibit more uniform behaviour patterns than local breeds (e.g., Grey Carmagnola Rabbit). In contrast, local breeds often display high exploratory and kinetic behaviours, along with greater behavioural variability, which may render them more responsive to environmental stimuli [30]. Furthermore, the same paper revealed the emergence of aggressive and dominance-related behaviours around 80–85 days of age, coinciding with the onset of sexual maturity. Contrarily, under our experimental conditions, rabbits did not exhibit aggressive behaviour, likely due to the high availability of space and the opportunity to express a broader behavioural repertoire [28], indirectly confirming the suitability of the proposed housing system.

The EFSA report [31] emphasised that housing in outdoor and organic rabbit farming systems can be highly diverse and complex. Therefore, this can result in different welfare consequences as fear (perceived exposure to predators) and health problems, which may result from exposure to thermal stress and limitations on biosecurity measures. Accordingly, hazard mitigation strategies must be tailored to each specific context. Under the experimental conditions adopted in the present study after one complete rearing cycle, no clinical signs of parasitosis were observed. However, it is important to note that coccidia counts may significantly increase in subsequent production cycles, necessitating ongoing monitoring and preventive measures [32].

The stocking density applied in our trial (5 m^2^ per rabbit) was equal to the first EU guidelines [16] and markedly higher than the minimum outdoor requirement established by Ministerial Decree 20 May 2022, which sets the threshold at just 0.5 m^2^ per rabbit. Based on our observations, mainly during the final rearing period, this standard appears absolutely insufficient not only for ensuring adequate forage intake of rabbits and mitigating health risks associated with high animal density, but also for enabling the full expression of the behavioural repertoire.

Previous papers showed that rabbits raised in outdoor conditions have shown greater physical activity than those raised indoors [33]. Having more space and environmental stimulation encourages them to move and explore. Exploration is an essential drive, both because it reflects the rabbit’s natural behavioral repertoire being a highly reactive and curious animal and because it relates to its instinct to search for food. The more space available, the greater the motivation for these animals to search for food; when food is no longer available in one area, they move elsewhere to look for it, which is why it is important to provide sufficient space [34]. Another key aspect is movement itself, which supports digestion, prevents musculoskeletal problems, strengthens the immune system, and improves product quality. Physical activity reduces issues linked to sedentary conditions, such as lameness, and pododermatitis. It also helps maintain strong muscles and a proper muscle-to-bone ratio. Overall, greater freedom of movement and the opportunity to express natural behaviours could improve animal welfare and the final product would also benefit, thanks to constant access to pastures rich in precursors for Polyunsaturated and other bioactive compounds [35].

In fact, although our trial was not specifically designed to quantify grass intake or assess nutritional self-sufficiency of growing rabbits, the estimation of forage availability provides approximately 40.5% and 36.1% of the total digestible energy in LV and NZW rabbits, respectively. According to several authors [36,37], a mean growth requirement of rabbits is met with 150 g DM/day. When offered both pasture and pelleted feed, rabbits typically consume at least half of their ration as pellets and the remainder as grass. Other studies [38,39] confirm that grass intake in outdoor-reared growing rabbits accounts for around 50% of total DM intake, influenced by live weight and pasture biomass availability. Naturally, the grass intake was also affected by season and optimal grass intake is achieved when biomass exceeds 3.5 t DM/ha, typical of natural spring pasture [39].

In this context, the current EU regulation for organic rabbit farming appears inconsistent. While it retains key recommendations, such as promoting pasture use and requiring a diet composed of at least 60% coarse fodder, it has relaxed several provisions. Notably, there is no longer a minimum slaughter age and rotational grazing is no longer mandatory. These changes, particularly those affecting pasture access and feeding practices, are likely to shape organic systems in ways that may reduce animal welfare and the nutritional adequacy of the system. Crucially, the regulations no longer require outdoor areas to be vegetated or used specifically for grazing. The previous two-month waiting period before reintroducing rabbits to grazed plots has also been eliminated. While these relaxations may simplify management, they increase the risk of parasitic exposure and undermine the agroecological approach of organic system. Given that a growing rabbit of about 2 kg, which in our trial is the average weight of animals at about 60–70 days of age, normally grazes between 0.5 and 1.0 m^2^ of pasture per day, the 0.5 m^2^ per rabbit stipulated by EU regulations would be depleted within 24 h. To meet the dietary requirement of 60% coarse fodder each rabbit would require nearly 1 m^2^ of high-quality pasture daily [32]. This stands in clear contradiction with Article 21 of Regulation (EU) 2020/464 [17], which mandates that: “The vegetation of the outdoor runs shall be maintained regularly and in such a way that it is attractive to rabbits” and “During the grazing season, pastures shall be rotated regularly and managed in such a way that the grazing of rabbits is optimised.”

As already mentioned in the paper, future research should address some limitations of this study, such as the limited number of replicates per breed, short study duration, and seasonal variability. As previously stated, the number of replicates represents a major constraint on the statistical power of the study; however, this limitation is inherent to research conducted in free-range systems, which require large land areas, complex management, and substantial economic resources, including intensive video-based monitoring of animals in outdoor environments. In these contexts, the use of three replicates was a compromise due to logistical, spatial and welfare constraints that are markedly different from those of intensive farming systems. Further investigations may provide external validation of our findings, which is necessary to confirm the observed behavioural dynamics and pasture utilisation both under similar environmental conditions and across different seasons.

Additional studies are recommended to explore long-term welfare outcomes, meat quality traits, and environmental interactions across diverse breeds, farming systems, and ecological conditions. Data collected during the spring–summer period, in particular, could be especially valuable for determining appropriate animal stocking densities throughout the year. Such insights would be crucial in refining density thresholds and developing effective pasture management strategies for the design of sustainable organic rabbit farming systems.

## 5. Conclusions

The results of this trial underscore the influence of genetic strain on behavioural patterns and pasture utilization in free range rabbit systems. Leprino di Viterbo demonstrated a higher frequency of grazing and active behaviours compared to New Zealand White rabbits, along with a greater reliance on grass intake despite similar overall feeding behaviour. These findings suggest that Leprino may be better adapted to outdoor conditions, particularly in systems that emphasize pasture-based nutrition and welfare. However, the observed decline in grass intake toward the end of the trial, recorded in both breeds and linked to reduced forage availability, highlights the importance of maintaining adequate pasture biomass throughout the rearing period.

Overall, this work provides exploratory insights into behavioural differences between rabbit breeds under outdoor conditions. The limited number of biological replicates should be carefully considered when interpreting these findings; nevertheless, the study highlights the importance of evaluating breed adaptability to alternative farming systems. This may contribute to a better understanding of potential strategies for optimising organic and free-range rabbit production systems. Further research is warranted to investigate breed-specific behavioural repertoires, seasonal variability in grazing dynamics, and their implications for animal welfare, feed efficiency and system sustainability.

## Figures and Tables

**Table 1:** Ingredients (%) and chemical composition (g/kg) of the solid feed and grass (mean + SD).

Ingredients	%	
Wheat bran	27.0	
Barley	21.0	
Hay	17.0	
Soybean meal	14.0	
Dehydrated alfalfa	8.0	
Sunflower meal	8.0	
Beet pulp	2.0	
Vegetable oil	1.0	
Vitamin and mineral supplement ^1^	1.0	
Calcium carbonate	0.5	
NaCl	0.5	
Chemical composition (g/kg)	Diet	Grass
Dry matter	882 ± 30	220 ± 10
Crude protein	164 ± 6	211 ± 8
Crude fibre	156 ± 8	331 ± 15
Ether extract	32.6 ± 1.5	183 ± 8
Ash	70.9 ± 2.9	81.1 ± 3.0
NDF	303 ± 12	585 ± 25
ADF	186 ± 8	328 ± 14
Hemicellulose	117 ± 5	273 ± 12
Cellulose	140 ± 6	220 ± 11
ADL	18.8 ± 1.0	76.4 ± 2.9
Digestible energy (DE) (kcal/kg) ^2^	2.450 ± 80	1.810 ± 71

^1^ kg: vit. A U.I. 11.000; vit. D_3_ U.I. 2.000; vit. B_1_ 2.5 mg; vit. B_2_ 4 mg; vit. B_6_ 1.25 mg; vit. B_12_ 0.01 mg; vit. E 25 mg; biotine 0.06 mg; vit. K 2.5 mg; niacine 15 mg; Folic ac. 0.30 mg; d-pantotenic ac. 10 mg; coline 600 mg; Mn 60 mg; Cu 3 mg; Fe 50 mg; Zn 15 mg; I 0.5 mg; Co 0.5 mg; lysine 50 mg; methionine 40 mg. ^2^ DE estimated with the following equation: (14.2 − 0.205 ADF + 0.218 EE + 0.057 CP) [26].

**Table 2:** Inventory of all the distinct, observable behaviours exhibited by rabbits.

Category	Behaviour	Description
Ingestive behaviours	Grazing	Eating grass directly from the ground, slowly and continuously
	Feeding	Consumption of feed supplied
	Drinking	Drink water from drinker
Active behaviours	Walking	Moving slowly on foot in a relaxed manner
	Running	Moving quickly with accelerated steps, often in response to stimulation
Total resting	Resting	Remaining still and relaxed, lying or standing quietly without sleeping
	Sleeping	Being asleep with closed eyes and a relaxed body posture
Reactive behaviours	Alert	Standing vigilant, head raised, ears forward, and attention directed to surroundings
	Hiding	Staying behind objects, vegetation, or in shelter to avoid being seen
Social behaviours	Allogrooming	Grooming, licking, or scratching another individual
	Stay close	Remaining near a conspecific to maintain social contact
	Aggressive	Chasing, biting, or showing dominance towards another rabbit
Miscellaneous	Self-grooming	Licking or scratching oneself to maintain cleanliness
	Stretching	Extending body or limbs to loosen muscles
	Digging	Using hooves, paws, or snout to move soil and create holes

## Data Availability

The data presented in this study are available on reasonable request from the corresponding author. The data are not publicly available due to institutional policy restrictions.

## Supplementary Materials

The following supporting information can be downloaded at: https://www.mdpi.com/article/10.3390/ani15243562/s1. Table S1. Overall means (and standard errors, SME) and marginal means (and SME) of behaviours according to breed and significance of effects assessed with statistical models. The behaviours are ordered from the highest to the lowest percentage. Only behaviours with a mean frequency of at least 1.0% in both breeds were included in the inferential analysis.
