# Supplementary material for: Outdoor Rearing and Behavioural Patterns in Diverse Rabbit Breeds: An Exploratory Study
Source: Animals (Basel). 2025 Dec 11;15(24):3562. doi: 10.3390/ani15243562 (PMC12729517; doi:10.3390/ani15243562)
Supplement: Supplementary file 1 [file animals-15-03562-s001.zip › animals-3997847-supplementary.pdf]

Supplementary Materials: Table S1. Overall means (and standard errors, SME) and marginal means (and SME) of behaviours according to genotype and significance of effects assessed with statistical models. The behaviours are ordered from the highest to the lowest percentage. Only behaviours with a mean frequency of at least 1.0% in both genotypes were included in the inferential analysis

Behaviour	Overall		Genotype				Significance			
			Leprino		New Zealand White					
	Mean	SEM	Mean	SEM	Mean	SEM	Genotype	Night/day	Time (age)	Time x genotype
**Resting**	26.22	1.80	9.59b	1.73	41.31a	2.64	<0.001	0.779	<0.001	<0.001
**Grazing**	23.78	1.87	35.37a	2.98	13.26b	2.07	<0.001	0.580	<0.001	<0.001
**Feeding**	20.12	1.85	19.60	2.68	20.58	2.55	0.828	0.644	<0.001	<0.001
**Walking**	8.64	0.93	11.01a	1.56	6.49b	1.06	<0.001	0.989	<0.001	<0.001
**Running**	7.25	0.97	13.95a	1.88	1.17b	0.39	<0.001	0.445	<0.001	<0.001
**Self-grooming**	5.85	0.87	5.66	1.25	6.02	1.22	0.687	0.934	<0.001	<0.001
**Stay close**	2.75	0.43	0.00b	0.00	5.24a	0.78	<0.001	0.038	<0.001	*
**Sleeping**	1.93	0.37	1.80	0.54	2.04	0.50	.946	0.256	<0.001	0.012
**Drinking**	1.04	0.39	0.00	0.00	1.98	0.75	0.082	0.167	<0.001	*
**Stretching**	0.75	0.29	1.45	0.60	0.12	0.12	-	-	-	-
**Allo-grooming**	0.67	0.29	0.88	0.58	0.48	0.19	-	-	-	-
**Alert**	0.48	0.20	0.64	0.38	0.34	0.16	-	-	-	
**Digging**	0.27	0.14	0.05	0.05	0.47	0.26	-	-	-	-
**Hiding**	0.26	0.26	0.00	0.00	0.49	0.49	-	-	-	-

•Unable to compute due to numerical problems
